# Establishing prognostic markers associated with neutrophil extracellular traps and the activated contact system in thrombotic microangiopathy

**DOI:** 10.1007/s00277-025-06612-7

**Published:** 2025-09-24

**Authors:** Su Sung Kim, Ja Yoon Gu, Sung Yoon Choi, Yujin Jung, Yoon Hwan Chang, Seon Young Kim, Hyun Kyung Kim

**Affiliations:** 1 Department of Laboratory Medicine, Seoul National University College of Medicine, Seoul, Republic of Korea; 2 Cancer Research Institute, Seoul National University College of Medicine, Seoul, Republic of Korea; 3 Department of Laboratory Medicine and Cancer Research Institute, Seoul National University College of Medicine, Seoul, Republic of Korea

**Keywords:** Thrombotic microangiopathy (TMA), Neutrophil extracellular traps (NETs), Contact system, Mortality, Prognostic marker

## Abstract

Thrombotic microangiopathy (TMA) is a life-threatening thrombotic disorder. Neutrophil extracellular traps (NETs) and activation of the contact system are known to promote and propagate the formation of thrombus. Our study assessed the prognostic values of multiple NET markers and a contact system activation marker in patients with clinically suspected TMA. Patients (*n* = 138) with clinically suspected TMA were analyzed for the circulating levels of NET markers (neutrophil elastase, histone-DNA complexes, citrullinated histone H3 [Cit H3], and cell-free double-stranded DNA [dsDNA]); the contact system activation marker (activated factor XII [factor XIIa]); and other TMA-associated markers (a disintegrin and metalloproteinase with a thrombospondin type 1 motif, member 13 [ADAMTS13] activity, von Willebrand factor antigen [vWF:Ag], von Willebrand factor ristocetin cofactor activity [vWF:Rco], and platelet counts). Low ADAMTS13 activity (hazard ratio [HR]: 4.85, *P* = 0.033), high vWF:Rco (HR: 9.46, *P* = 0.037), low platelet counts (HR: 7.45, *P* = 0.017), and high histone-DNA complexes (HR: 8.43, *P* = 0.015) were identified as independent markers of high mortality in multivariable Cox proportional hazards regression analysis. ADAMTS13, vWF:Rco, platelet counts, and histone-DNA complexes can be useful markers for identifying TMA patients at high risk of mortality.

**Supplementary Information:**

The online version contains supplementary material available at 10.1007/s00277-025-06612-7.

## Introduction

Thrombotic microangiopathy (TMA) is a life-threatening thrombotic disorder marked by microangiopathic hemolytic anemia (MAHA), thrombocytopenia, and subsequent end-organ dysfunction [1]. TMA can be classified into primary and secondary subtypes based on the underlying etiology [2, 3]. A severe deficiency of ADAMTS13, a metalloprotease that cleaves ultra-large von Willebrand factor (vWF) multimers, causes thrombotic thrombocytopenic purpura (TTP) that is a subtype of primary TMA [2]. Several studies have investigated the prognostic factors associated with TTP [4, 5]. To ensure prompt and appropriate management of patients with suspected TMA in clinical practice, an early assessment is desirable before a defined subtype diagnosis. Hence, there is a need for reliable prognostic markers that can be applied across the TMA spectrum, but these markers have not yet been clearly established.

During inflammation, neutrophils release their nuclear contents into the extracellular space to form neutrophil extracellular traps (NETs) to capture bacteria or to provide a scaffold for the hemostatic plug of thrombosis. NETs are composed of histone-DNA complexes and serine proteases including neutrophil elastase [6]. Since platelets adhere to the scaffold of NETs, extensive NET formation promotes the development of thrombosis [6]. Although there have been reports of several TMA patients with high levels of NET markers [7], the prognostic value of NET markers in TMA has not yet been investigated.

The negatively-charged surface of DNA released from NETs can activate the contact system that includes coagulation factors XII and XI in the intrinsic coagulation pathway [6]. Since triggering this pathway can promote thrombosis, contact system activation has been reported in thrombo-inflammatory disorders, such as disseminated intravascular coagulation (DIC) and sickle cell disease [8, 9]. To date, activation of the contact system has not been clearly demonstrated in TMA.

In this study, we comprehensively evaluated the prognostic value of NET markers and a contact system activation marker in relation to 30-day mortality across the entire spectrum of clinically suspected TMA. We measured the following: (1) circulating levels of four NET markers (neutrophil elastase, histone-DNA complexes, citrullinated histone H3 [Cit H3], and cell-free double-stranded DNA [dsDNA]); (2) a contact system activation marker (activated factor XII [factor XIIa]); and (3) other TMA-associated markers (a disintegrin and metalloproteinase with a thrombospondin type 1 motif, member 13 [ADAMTS13] activity, von Willebrand factor antigen [vWF:Ag], von Willebrand factor ristocetin cofactor activity [vWF:Rco], and platelet counts).

## Methods

### Study population

This single-center study included 154 subjects who underwent testing for ADAMTS13 activity due to clinically suspected TMA between May 24, 2016 and June 10, 2021. Of the 154 patients, 16 patients were excluded since they had already received plasma-based therapies such as plasma exchange or plasma transfusion prior to testing. Therefore, a total of 138 patients were included in the final analysis (Supplementary Material 1, Fig. S1). TMA was suspected in patients presenting with both acute thrombocytopenia—defined as a platelet count < 150 × 10⁹/L or a significant decrease (> 50%) from baseline—and MAHA, as evidenced by the presence of schistocytes (> 1%) on a peripheral blood smear and/or laboratory markers of hemolysis, including elevated lactate dehydrogenase, decreased haptoglobin, and increased indirect bilirubin. Clinical and laboratory data were retrospectively collected through electronic medical records. The Institutional Review Board (IRB) of Seoul National University College of Medicine approved the study protocol.

### Marker analysis

ADAMTS13 activity and anti-ADAMTS13 antibody were measured using ELISA-based kits (Technozym^®^ ADAMTS13 Activity and INH assays, Technoclone, Vienna, Austria). A positive cutoff for anti-ADAMTS13 antibody was 15 U/mL, as recommended by the manufacturer. The REAADS vWF:Ag kit (Corgenix, Westminster, CO, USA) measured vWF:Ag, and the Innovance vWF Ac Assay (OPHL03, Siemens, Quebec, Canada) measured vWF:Rco on an IL ACL 3000 coagulation analyzer (Werfen, Barcelona, Spain), respectively. The levels of histone-DNA complexes were measured using the Cell Death Detection ELISA kit (Roche Diagnostics, Indiana, USA). A chromogenic assay (CoaChrom Diagnostica, Maria Enzersdorf, Austria) was used to measure the activity of factor XIIa. Neutrophil elastase levels were measured using a Human PMN Elastase Platinum ELISA kit (eBioscience, Vienna, Austria). Cit H3 levels were measured with a sandwich ELISA kit (Citrullinated Histone H3 ELISA Kit, Clone 11D3; Cayman Chemical, Ann Arbor, MI, USA). Cell-free dsDNA was measured using a fluorescence-based assay with the Quant-iT™ PicoGreen^®^ dsDNA reagent (Molecular Probes, Eugene, OR, USA). All the markers were measured according to the respective manufacturer’s instructions, using blood samples collected at the time of ADAMTS13 activity testing in patients with clinically suspected TMA who had not received prior plasma-based therapies.

### Statistical analysis

The Mann-Whitney U test was used to compare continuous variables of the two groups. The Chi-square test or Fisher’s exact test was used to compare the categorical variables. The prognostic value of each marker was assessed with Cox proportional hazards regression analyses with stepwise selection based on Akaike Information Criterion. Receiver operating characteristic (ROC) curves identify optimal cutoff values that are used for Kaplan-Meier survival analyses and Cox proportional hazards regression analyses of the variables associated with the 30-day survival status. The cutoff values determined by ROC analysis represent optimal discriminatory thresholds from a statistical perspective and thus may differ from clinically established or practically meaningful values. The Statistical Package for the Social Sciences (version 29.0; IBM Corp., Armonk, NY, USA) and the R software (version 4.4.0; R Core Team, Vienna, Austria) were used to perform statistical analyses. A *P*-value < 0.05 was considered statistically significant.

## Results

### Baseline characteristics

A total of 138 patients were classified into two groups according to their 30-day survival status (123 survivors, 15 non-survivors; Table 1). Although non-survivors were significantly older, there was no significant difference in the distribution of clinical diagnoses between the two groups. There was a marked decrease in ADAMTS13 activity and platelet counts with slightly elevated vWF:Ag levels in the non-survivor group.

**Table 1:** Baseline characteristics and the values of markers in the study population (*n* = 138) classified based on the 30-day survival status

Characteristics	Survivors (*n* = 123)		Non-survivors (*n* = 15)		*P*-value
Age (years)	57.0	(36.0–67.0)	71.0	(65.5–74.5)	< 0.001
Sex, male/female	61 (49.6)	/62 (50.4)	7 (46.7)	/8 (53.3)	0.831
Clinical diagnosis					
Solid tumors	13	(10.6)	3	(20.0)	0.383
Hematologic malignancy	14	(11.4)	3	(20.0)	0.398
Solid transplantation	47	(38.2)	2	(13.3)	0.057
Hematologic transplantation	10	(8.1)	2	(13.3)	0.620
Platelets (× 10^9^/L)	76	(37–115)	36	(27–43)	0.004
Fibrinogen (mg/dL)	281	(220–373)	287	(215–355)	0.816
D-dimer (ng/mL)	848	(424–1333)	1289	(750–2240)	0.055
ADAMTS13 activity (%)	67	(53–84)	46	(35–59)	0.002
Severe ADAMTS13 deficiency^*^	3	(2.5)	1	(6.7)	0.379
Anti-ADAMTS13 Ab positivity^†^	2	(1.7)	1	(7.1)	0.288
vWF:Ag (%)	231.3	(194.8–323.8)	305.2	(227.9–535.9)	0.042
vWF:Rco (%)	158.7	(113.4–242.9)	204.8	(165.0–335.4)	0.069
Factor XIIa (U/L)	40.1	(28.8–61.4)	38.1	(27.8–58.8)	0.980
Neutrophil elastase (ng/mL)	57.0	(30.5–127.0)	40.0	(29.9–97.3)	0.354
Histone-DNA complexes (AU)	57	(34–116)	64	(46–159)	0.636
Cit H3 (ng/mL)	8.24	(4.01–13.57)	5.31	(3.11–9.08)	0.236
Cell-free dsDNA (ng/mL)	75.3	(65.3–94.0)	72.8	(60.2–101.3)	0.528

Values are presented as number of subjects (percentage) or median (interquartile range)^*^Severe ADAMTS13 deficiency indicates that the ADAMTS13 activity is less than 10%. Data on ADAMTS13 activity were missing in 3 survivors^†^Anti-ADAMTS13 Ab positivity indicates that the anti-ADAMTS13 antibody exceeded 15 U/mL. Data on anti-ADAMTS13 antibody were missing in 5 survivors and 1 non-survivor. Abbreviations: *Ab *antibody, *AU* arbitrary units, *ADAMTS13* a disintegrin and metalloproteinase with a thrombospondin type 1 motif, member 13, *vWF* von Willebrand factor, *Ag* antigen, *Rco* ristocetin cofactor, *Cit H3* citrullinated histone H3, *Cell-free dsDNA* cell-free double-stranded deoxyribonucleic acid

Among the 138 patients in the study cohort, 4 patients (3 survivors and 1 non-survivor) were identified with severe ADAMTS13 deficiency (< 10%), consistent with a diagnosis of TTP (Supplementary Material 1, Supplementary Table 1). Of these, 2 patients had elevated anti-ADAMTS13 antibody levels, indicating immune-mediated TTP. The remaining 2 patients were suspected of congenital TTP, although genetic testing was not performed and family history was not clearly documented.

An additional 4 patients (4 survivors) were diagnosed with infection-associated hemolytic-uremic syndrome (IA-HUS) based on the detection of STX1 or STX2 genes using Polymerase Chain Reaction (PCR) testing for enterohemorrhagic *Escherichia coli* (EHEC) from stool specimens. 5 patients (2 survivors and 3 non-survivors) were diagnosed with DIC, defined by a score ≥ 5 according to the 2025 International Society on Thrombosis and Haemostasis (ISTH) criteria [10].

Additionally, 16 patients (12 survivors and 4 non-survivors) were classified as malignancy-related TMA, and 35 patients (34 survivors and 1 non-survivor) were classified as transplant-related TMA, both based on their medical history. 47 patients (43 survivors and 4 non-survivors) were classified as presumptive atypical HUS, defined by renal abnormalities such as significantly elevated creatinine levels, absence of criteria for secondary TMA, negative EHEC PCR test results, and normal ADAMTS13 activity. The remaining 27 patients (25 survivors and 2 non-survivors) were classified as other forms of TMA.

Patients diagnosed with specific diseases received treatment as determined by their clinicians. Those with TTP underwent therapeutic plasma exchange; those with IA-HUS received supportive care; and those with malignancy-related or transplant-related TMA were treated according to their underlying diseases. Patients with presumptive atypical HUS received supportive care, and eculizumab was also considered a potential treatment option.

### Prognostic values of markers

**Fig. 1:**
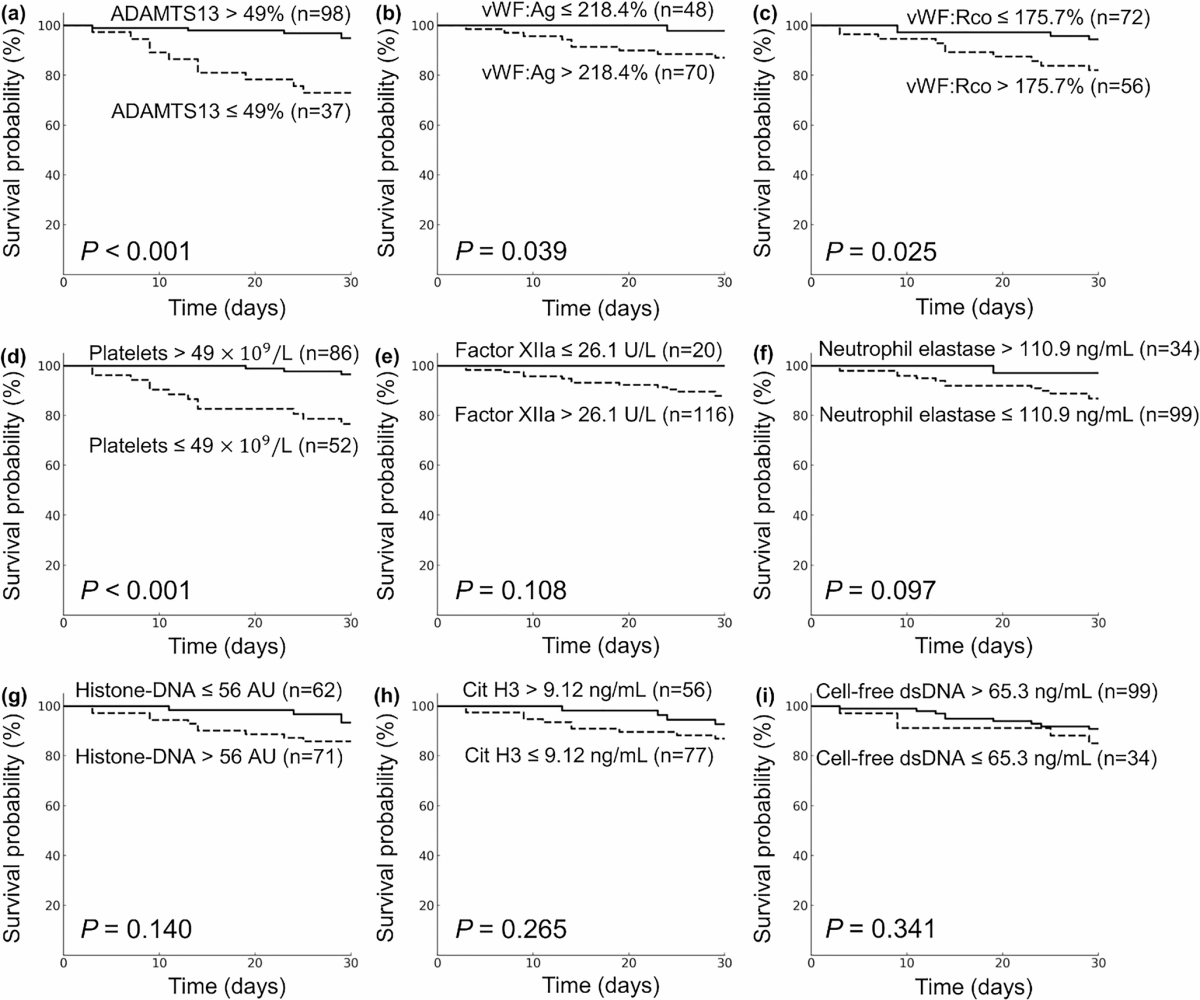
Kaplan-Meier survival curves. Patients were divided into survivors and non-survivors according to the indicated cutoff values of (**a**) ADAMTS13 activity, (**b**) vWF:Ag, (**c**) vWF:Rco, (**d**) Platelets, (**e**) Factor XIIa, (**f**) Neutrophil elastase, (**g**) Histone-DNA complexes, (**h**) Cit H3, and (**i**) Cell-free dsDNA

Kaplan-Meier analyses stratified patients into two groups – survivors and non-survivors – based on optimal cutoff values determined by ROC analysis (Fig. 1). Patients with low ADAMTS13 activity (≤ 49%) exhibited significantly higher mortality than those with high ADAMTS13 activity (> 49%) (*P* < 0.001; Fig. 1a). High levels of vWF:Ag (> 218.4%) and vWF:Rco (> 175.7%) were also significantly associated with higher mortality (*P* = 0.039 and *P* = 0.025, respectively; Fig. 1b and c). Similarly, low platelet counts (≤ 49 × 10^9^/L) were associated with higher mortality (*P* < 0.001; Fig. 1d). However, factor XIIa and NET markers including neutrophil elastase, histone-DNA complexes, Cit H3, and cell-free dsDNA did not show statistically significant differences in mortality between survivor and non-survivor groups (Fig. 1e–i).

In univariable Cox proportional hazards regression analysis (Table 2), ADAMTS13 activity demonstrated a significant hazard ratio (HR: 6.03, *P* = 0.001). Significant hazard ratios were also identified for vWF:Rco (HR: 3.46, *P* = 0.036) and platelet counts (HR: 7.61, *P* = 0.002). In the multivariable Cox proportional hazards regression analysis (Table 2), independent prognostic markers were identified by the hazard ratios of ADAMTS13 activity (HR: 4.86, *P* = 0.033), vWF:Rco (HR: 9.46, *P* = 0.037), platelet counts (HR: 7.45, *P* = 0.017), and histone-DNA complexes (HR: 8.43, *P* = 0.015).

**Table 2:** Cox regression analyses of circulating markers to predict mortality

Variables	Univariable			Multivariable		
	HR	95% CI	*P*-value	HR	95% CI	*P*-value
ADAMTS13 activity (> 49 vs. ≤ 49%)	6.03	2.1–17.7	0.001	4.86	1.1–20.8	0.033
vWF:Ag (≤ 218.4 vs. > 218.4%)	6.56	0.8–51.8	0.074			
vWF:Rco (≤ 175.7 vs. > 175.7%)	3.46	1.9–11.0	0.036	9.46	1.1–78.0	0.037
Platelets (> 49 vs. ≤ 49 × 10^9^/L)	7.61	2.1–27.0	0.002	7.45	1.4–38.9	0.017
Factor XIIa (≤ 26.06 vs. > 26.06 U/L)	0.19	0.0–1.4	0.124			
Neutrophil elastase (> 110.9 vs. ≤ 110.9 ng/mL)	4.74	0.6–36.2	0.134			
Histone-DNA complexes (≤ 56 vs. > 56 AU)	2.33	0.7–7.4	0.152	8.43	1.5–47.6	0.015
Cit H3 (> 9.12 vs. ≤ 9.12 ng/mL)	1.91	0.6–6.1	0.274			
Cell-free dsDNA (> 65.25 vs. ≤ 65.25 ng/mL)	1.69	0.6–5.0	0.347			

Abbreviations: *AU *arbitrary units, *ADAMTS13 *a disintegrin and metalloproteinase with a thrombospondin type 1 motif, member 13, *vWF *von Willebrand factor, *Ag *antigen, *Rco *ristocetin cofactor, *Cit H3 *citrullinated histone H3, *Cell-free dsDNA *cell-free double-stranded deoxyribonucleic acid, *HR *hazard ratio, *CI *confidence interval

### Subgroup analyses

To enhance the clinical relevance of our findings, we conducted subgroup analyses for malignancy-related TMA (*n* = 16) and transplant-related TMA (*n* = 35). In the malignancy-related TMA group, non-survivors were significantly older (*P* = 0.030) and showed a trend toward lower platelet counts (*P* = 0.078) than survivors (Supplementary Material 1, Supplementary Table 2). Kaplan-Meier analyses, using the same cutoff values as in the total cohort, showed trends toward higher mortality in patients with low ADAMTS13 activity (≤ 49%) and low platelet counts (≤ 49 × 10^9^/L) (both *P* = 0.051; Supplementary Material 1, Fig. S2a, S2d). In the transplant-related TMA group, no variables reached statistical significance in the Mann-Whitney U test (Supplementary Material 1, Supplementary Table 3), but low ADAMTS13 activity (≤ 49%) was significantly associated with higher mortality (*P* = 0.031; Supplementary Material 1, Fig. S3a). In both subgroups, Cox proportional hazards regression analyses revealed no statistically significant predictors, likely due to the limited number of events and highly skewed value distributions (Supplementary Material 1, Supplementary Tables 4–5).

## Discussion

This study identified several key prognostic markers associated with 30-day mortality in patients with clinically suspected TMA: (1) Low circulating levels of ADAMTS13, (2) High levels of vWF:Rco, (3) Low platelet counts, and (4) High levels of histone-DNA complexes, a component of NETs. These findings suggest that specific hemostatic and inflammatory markers may provide early prognostic information in the management of patients with clinically suspected TMA.

In clinical practice, the majority of TMA patients are classified as secondary TMA due to underlying comorbidities, while primary TMA, such as TTP, is rare [11]. Although prompt and accurate classification of TMA is essential to determine appropriate treatment, it is challenging due to the diversity of the subtypes of TMA and the limitations in the routine use of genetic tests such as EHEC PCR in clinical practice. Therefore, there is an increasing need for novel markers that can predict the mortality of patients with clinically suspected TMA prior to definitive diagnosis.

Using multivariable Cox proportional hazards regression analysis to identify prognostic markers in TMA, we found that low ADAMTS13 activity was significantly associated with high mortality in patients with clinically suspected TMA (Table 2). ADAMTS13 deficiency leads to the accumulation of ultra-large vWF multimers that promote excessive platelet adhesion and aggregation [2]. This results in the widespread formation of microvascular thrombi, causing tissue ischemia and multiorgan failure [2].

ADAMTS13 deficiency can also lead to the accumulation of NETs and promote complement activation on endothelial surfaces, which contribute to systemic inflammation and thrombotic injury in TTP [12, 13]. Recent studies have also reported that NET formation and ADAMTS13 deficiency similarly contribute to thrombosis and inflammation in other thrombo-inflammatory conditions, such as DIC and sickle cell disease [8, 9], further supporting our findings. Although the ADAMTS13 deficiency in TTP is mainly caused by anti-ADAMTS13 antibody, the mild deficiency of ADAMTS13 in a variety of conditions including sepsis, infection and other subtypes of TMA may be caused by the processing of ADAMTS13 through the continuous cleavage of vWF in microthrombi during the inflammatory process [14, 15]. Therefore, ongoing extensive thrombus formation continues to reduce the levels of ADAMTS13, and the reduced ADAMTS13 activity could be used as a prognostic marker in TMA.

In our study, high levels of vWF:Rco were significantly associated with high mortality, whereas vWF:Ag levels showed no significant association (Table 2). Endothelial cells mainly produce vWF that promotes platelet adhesion and aggregation at sites of injury by binding to collagen and platelet receptors, playing a key role in hemostasis and thrombosis [1]. vWF:Rco reflects the ability of vWF to bind platelet glycoprotein Ib receptor in the presence of ristocetin. The ability of vWF to bind depends on high-molecular-weight multimers and makes it sensitive to qualitative vWF abnormalities [16]. In contrast, vWF:Ag reflects the total amount of vWF protein present in plasma. High levels of vWF:Rco indicate increased platelet aggregation and microvascular thrombosis. Therefore, vWF:Rco may serve as a more sensitive prognostic marker than vWF:Ag in TMA.

Low platelet counts in our results were significantly associated with high mortality in patients with clinically suspected TMA (Table 2). A similar observation has also been reported in patients with TMA secondary to systemic lupus erythematosus [17]. Severe thrombocytopenia likely reflects the degree of ongoing microvascular thrombosis and resultant organ damage, both of which are key determinants of high mortality in TMA.

Although factor XIIa did not demonstrate prognostic value for 30-day mortality in our analysis (Table 2), we could not adequately evaluate its diagnostic value due to the limited number of TTP patients included in this study. Thus, future prospective studies specifically designed to assess the diagnostic potential of factor XIIa for TTP or contact system activation are warranted.

There was a significant association of high levels of histone-DNA complexes, a key component of NETs, with high mortality in patients with clinically suspected TMA (Table 2). NETs are web-like interlinked structures of DNA, histones, and antimicrobial proteins released by neutrophils to trap and eliminate pathogens [6]. NET formation contributes to endothelial damage through the release of cytotoxic components, thereby exacerbating intravascular thrombosis that is associated with disease [7]. The high levels of histone-DNA complexes suggest that such mechanisms are also involved in the pathophysiology of TMA and may lead to high mortality. In contrast, other NET markers, including neutrophil elastase, Cit H3, and cell-free dsDNA, did not demonstrate significant differences in relation to mortality (Table 2). These findings suggest that histone-DNA complexes may serve as a more sensitive prognostic marker than other NET markers in TMA [18]. This may be due to underlying biological differences or to differences in assay sensitivity, specificity, and stability among detection methods.

Our study has several limitations. First, it was conducted at a single center with a small sample size. Consequently, we were unable to identify the prognostic markers for individual TMA subtypes despite performing subgroup analyses for malignancy-related and transplant-related TMA. Both subgroups exhibited patterns similar to the total cohort, but no significant predictors were identified by Cox regression, likely due to the small number of events and insufficient statistical power. These findings warrant validation in larger, subtype-focused studies. In addition, TMA subtypes were not clearly defined in our study population. Although our findings may be useful in general clinical practice to assess patients with overall TMA, further large-scale studies are required to confirm their applicability to specific subtypes. Second, this study was retrospective in design and used real-world clinical data, limiting our ability to evaluate outcomes by specific treatment modalities because of the heterogeneity of therapeutic approaches across subtypes. Accordingly, prospective studies that comprehensively assess various therapeutic interventions are needed.

In conclusion, our findings highlight the potential of low circulating levels of ADAMTS13, high levels of vWF:Rco, low platelet counts, and high levels of histone-DNA complexes to support early prognostic assessment in TMA prior to definitive diagnosis. These markers may help guide clinical decision-making in patients with clinically suspected TMA. However, validation in large, prospective, subtype-defined cohorts is essential before clinical implementation.

## Supplementary Information

Below is the link to the electronic supplementary material.

Supplementary Material 1 (PDF 702 KB)

## Data Availability

This study used patient data from the Seoul National University Hospital (SNUH) electronic medical records, which are not publicly available due to privacy regulations. The data may be available from the corresponding author upon reasonable request and with appropriate institutional approval.
