# Supplementary material for: Establishing prognostic markers associated with neutrophil extracellular traps and the activated contact system in thrombotic microangiopathy
Source: Ann Hematol. 2025 Sep 24;104(10):5433–9. doi: 10.1007/s00277-025-06612-7 (PMC12619824; doi:10.1007/s00277-025-06612-7)
Supplement: Supplementary file 1 — Supplementary Material 1 (PDF 702 KB) [file 277_2025_6612_MOESM1_ESM.pdf]

Establishing Prognostic Markers Associated with Neutrophil Extracellular Traps and the Activated Contact System in Thrombotic Microangiopathy

Running Title: Prognostic markers in TMA

Authors

Su Sung Kim[1], Ja Yoon Gu[2,3], Sung Yoon Choi[3], Yujin Jung[1], Yoon Hwan Chang[2], Seon Young Kim[2], Hyun Kyung Kim[2,3]

Affiliations

[1]Seoul National University College of Medicine, Seoul, Republic of Korea

[2]Department of Laboratory Medicine, Seoul National University College of Medicine, Seoul, Republic of Korea

[3]Cancer Research Institute, Seoul National University College of Medicine, Seoul, Republic of Korea

Corresponding Author

Correspondence to Hyun Kyung Kim, MD, PhD, Department of Laboratory Medicine and Cancer Research Institute, Seoul National University College of Medicine, 101, Daehak-ro, Jongno-gu, Seoul 03080, Republic of Korea, Tel: +82-2-2072-0853, Fax: +82-2-747-0359, Email: lukekhk@snu.ac.kr, ORCID ID: https://orcid.org/0000-0003-2401-3933

Supplementary Table 1 Diagnostic classification of thrombotic microangiopathies in total enrolled patients

Diagnosis	Total (n=138)	Survivors (n=123)	Non-survivors (n=15)
TTP	4 (2.9)	3 (75.0)	1 (25.0)
Congenital	2 (1.4)	1 (50.0)	1 (50.0)
Immune-mediated	2 (1.4)	2 (100.0)	0 (0.0)
Malignancy-related TMA	16 (11.6)	12 (75.0)	4 (25.0)
Transplant-related TMA	35 (25.4)	34 (97.1)	1 (2.9)
Infection-associated HUS	4 (2.9)	4 (100.0)	0 (0.0)
Presumptive atypical HUS	47 (34.1)	43 (91.5)	4 (8.5)
DIC	5 (3.6)	2 (40.0)	3 (60.0)
Others	27 (19.6)	25 (92.6)	2 (7.4)

Values are presented as number of subjects (percentage).
Abbreviations: TMA - thrombotic microangiopathy; TTP - thrombotic thrombocytopenic purpura; HUS - hemolytic-uremic syndrome; DIC - disseminated intravascular coagulation

Supplementary Table 2 Baseline characteristics and the values of markers in the malignancy-related TMA population (n = 16) classified based on the 30-day survival status

**Characteristics**	**Survivors (n=12)**	**Non-survivors (n=4)**	***P*-value**
Age (years)	60.5 (50.5–65.5)	69.5 (65.8–83.8)	0.030
Sex, male/female	8 (66.7) / 4 (33.3)	2 (50.0) / 2 (50.0)	0.604
Platelets (× 10^9/L)	62 (21–87)	21 (9–33)	0.078
Fibrinogen (mg/dL)	224 (199–316)	355 (243–355)*	0.170
D-dimer (ng/mL)	937 (845–2007)	566 (464–566)*	0.073
ADAMTS13 activity (%)	53 (34–86)	44 (29–46)	0.262
vWF:Ag (%)	222.6 (200.5–308.4)	457.0 (325.0–457.0)*	0.089
vWF:Rco (%)	140.3 (106.1–260.7)	270.1 (131.0–370.0)	0.304
Factor XIIa (U/L)	52.1 (36.2–69.6)	39.0 (28.0–56.4)	0.212
Neutrophil elastase (ng/mL)	49.2 (27.7–89.5)	26.2 (19.7–26.2)*	0.233
Histone-DNA (AU)	59.5 (31.8–242.8)	59.0 (58.0–59.0)*	0.734
Cit H3 (ng/mL)	7.23 (3.70–16.95)	4.75 (1.28–4.75)*	0.365
Cell-free dsDNA (ng/mL)	82.9 (58.9–150.1)	35.0 (24.2–35.0)*	0.233

Values are presented as number of subjects (percentage) or median (interquartile range).

*Due to the small sample size in the non-survivor group, some quartile values are identical to the median.

Abbreviations: AU - arbitrary units; ADAMTS13 - a disintegrin and metalloproteinase with a thrombospondin type 1 motif, member 13; vWF - von Willebrand factor; Ag - antigen; Rco - ristocetin cofactor; Cit H3 - citrullinated histone H3; Cell-free dsDNA - cell-free double-stranded deoxyribonucleic acid

Supplementary Table 3 Baseline characteristics and the values of markers in the transplant-related TMA population (n = 35) classified based on the 30-day survival status

**Characteristics**	**Survivors (n=34)**	**Non-survivor[†] (n=1)**	***P*-value**
Age (years)	52.5 (38.0–65.3)	73.0 (73.0–73.0)[*]	0.114
Sex, male/female	24 (70.6) / 10 (29.4)	0 (0.0) / 1 (100.0)	0.314
Platelets (× 10^9/L)	82 (39–121)	32 (32–32)[*]	0.400
Fibrinogen (mg/dL)	257 (214–319)		
D-dimer (ng/mL)	533 (284–1116)		
ADAMTS13 activity (%)	67 (55–93)	49 (49–49)[*]	0.353
vWF:Ag (%)	235.0 (192.0–396.8)		
vWF:Rco (%)	148.0 (113.1–295.9)		
Factor XIIa (U/L)	37.8 (28.7–53.1)		
Neutrophil elastase (ng/mL)	59.9 (28.8–173.9)	88.2 (88.2–88.2)[*]	0.848
Histone-DNA (AU)	65.5 (41.3–126.0)	3.0 (3.0–3.0)[*]	0.061
Cit H3 (ng/mL)	7.41 (3.53–13.61)	2.52 (2.52–2.52)[*]	0.364
Cell-free dsDNA (ng/mL)	72.0 (64.1–87.5)	65.3 (65.3–65.3)[*]	0.545

Values are presented as number of subjects (percentage) or median (interquartile range).
[*]Quartile values in the non-survivor group are identical to the median because only one observation was available.
[†]Data for fibrinogen, D-dimer, vWF:Ag, vWF:Rco, and Factor XIIa were missing in the non-survivor group and thus excluded from the analysis.
Abbreviations: AU - arbitrary units; ADAMTS13 - a disintegrin and metalloproteinase with a thrombospondin type 1 motif, member 13; vWF - von Willebrand factor; Ag - antigen; Rco - ristocetin cofactor; Cit H3 - citrullinated histone H3; Cell-free dsDNA - cell-free double-stranded deoxyribonucleic acid

Supplementary Table 4 Cox regression analyses of circulating markers to predict mortality in the malignancy-related TMA population

Variables	Univariable			Multivariable†		
	HR	95% CI	*P*-value	HR	95% CI	*P*-value
ADAMTS13 activity (> 49 vs. ≤ 49%)	62.62	0.0–2.0×10^5	0.314			
vWF:Ag (≤ 218.4 vs. > 218.4%)	36.92	0.0–1.0×10^7	0.572			
vWF:Rco (≤ 175.7 vs. > 175.7%)	3.56	0.4–34.4	0.272			
Platelets (> 49 vs. ≤ 49 × 10^9/L)	62.62	0.0–2.0×10^5	0.314			
Factor XIIa (≤ 26.06 vs. > 26.06 U/L)*						
Neutrophil elastase (> 110.9 vs. ≤ 110.9 ng/mL)	22.50	0.0–5.9×10^9	0.753			
Histone-DNA complexes (≤ 56 vs. > 56 AU)	51.07	0.0–6.4×10^5	0.414			
Cit H3 (> 9.12 vs. ≤ 9.12 ng/mL)	41.20	0.0–7.7×10^5	0.459			
Cell-free dsDNA (> 65.25 vs. ≤ 65.25 ng/mL)	3.67	0.3–40.7	0.290			

*Factor XIIa was not analyzed because all patients were in the same category.
†Multivariable analysis did not yield a model because the model did not converge due to an insufficient number of events relative to the number of variables, combined with complete separation in several predictors.
Abbreviations: AU - arbitrary units; ADAMTS13 - a disintegrin and metalloproteinase with a thrombospondin type 1 motif, member 13; vWF - von Willebrand factor; Ag - antigen; Rco - ristocetin cofactor; Cit H3 - citrullinated histone H3; Cell-free dsDNA - cell-free double-stranded deoxyribonucleic acid; HR - hazard ratio; CI - confidence interval

Supplementary Table 5 Cox regression analyses of circulating markers to predict mortality in the transplant-related TMA population

**Variables**	**Univariable**			**Multivariable**[†]		
	**HR**	**95% CI**	***P*-value**	**HR**	**95% CI**	***P*-value**
ADAMTS13 activity (> 49 vs. ≤ 49%)	2.18×10^{3}	$0.0–6.2\times10^{21}$	0.723			
vWF:Ag (≤ 218.4 vs. > 218.4%)*						
vWF:Rco (≤ 175.7 vs. > 175.7%)*						
Platelets (> 49 vs. ≤ 49 × 10^{9}/L)	2.01×10^{2}	$0.0–3.6\times10^{10}$	0.585			
Factor XIIa (≤ 26.06 vs. > 26.06 U/L)*						
Neutrophil elastase (> 110.9 vs. ≤ 110.9 ng/mL)	45.40	$0.0–7.5\times10^{9}$	0.653			
Histone-DNA complexes (≤ 56 vs. > 56 AU)	0.01	$0.0–1.4\times10^{5}$	0.596			
Cit H3 (> 9.12 vs. ≤ 9.12 ng/mL)	45.40	$0.0–7.5\times10^{9}$	0.653			
Cell-free dsDNA (> 65.25 vs. ≤ 65.25 ng/mL)	0.03	$0.0–9.4\times10^{6}$	0.730			

*vWF:Ag, vWF:Rco, and Factor XIIa were not analyzed because all patients were in the same category.
†Multivariable analysis did not yield a model because the model did not converge due to an insufficient number of events relative to the number of variables, combined with complete separation in several predictors.

Abbreviations: AU - arbitrary units; ADAMTS13 - a disintegrin and metalloproteinase with a thrombospondin type 1 motif, member 13; vWF - von Willebrand factor; Ag - antigen; Rco - ristocetin cofactor; Cit H3 - citrullinated histone H3; Cell-free dsDNA - cell-free double-stranded deoxyribonucleic acid; HR - hazard ratio; CI - confidence interval

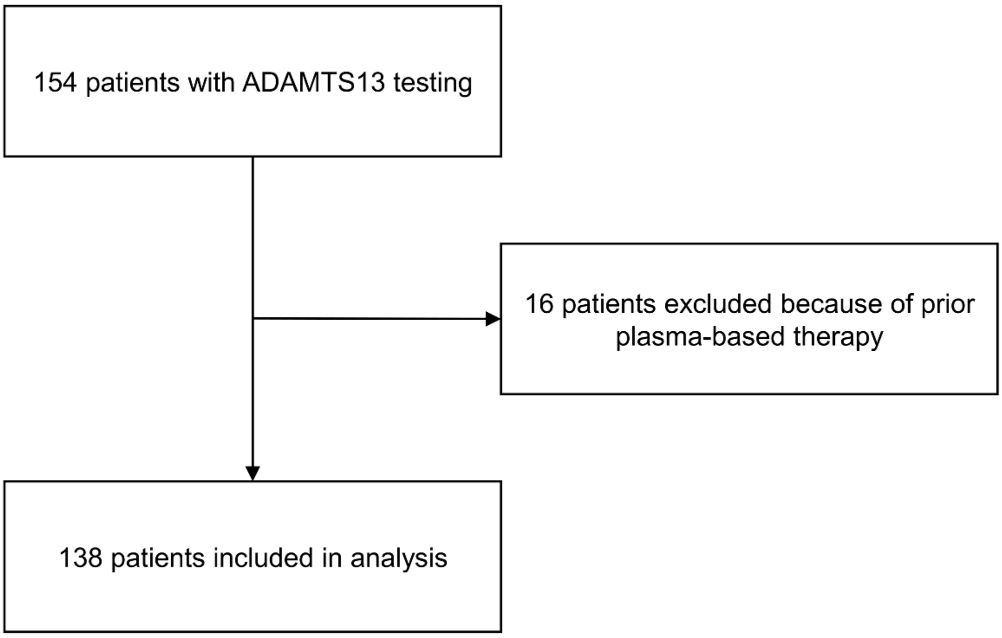

Fig. S1 Flow diagram of patient selection for the final analysis

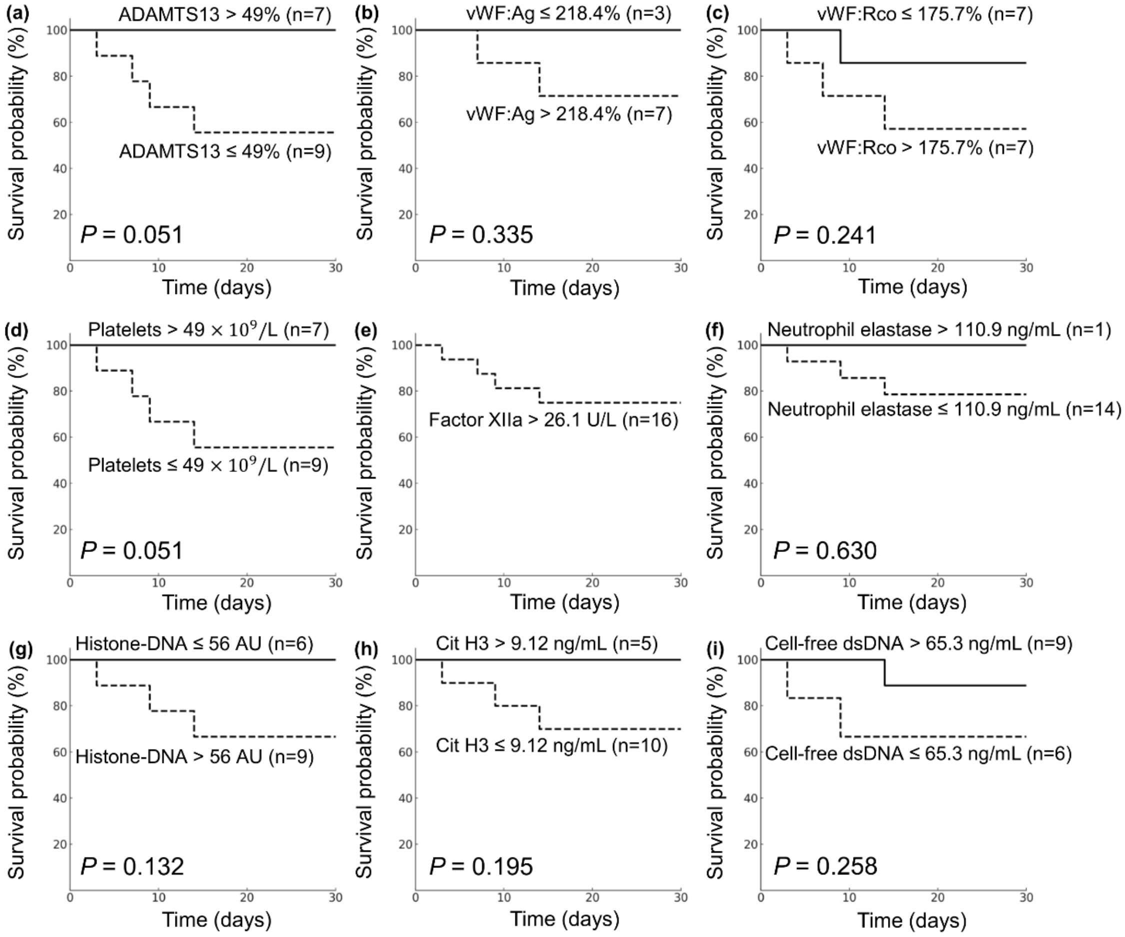

Fig. S2 Kaplan-Meier survival curves in the malignancy-related TMA population. Patients were divided into survivors and non-survivors according to the indicated cutoff values of (a) ADAMTS13 activity, (b) vWF:Ag, (c) vWF:Rco, (d) Platelets, (e) Factor XIIa, (f) Neutrophil elastase, (g) Histone-DNA complexes, (h) Cit H3, and (i) Cell-free dsDNA

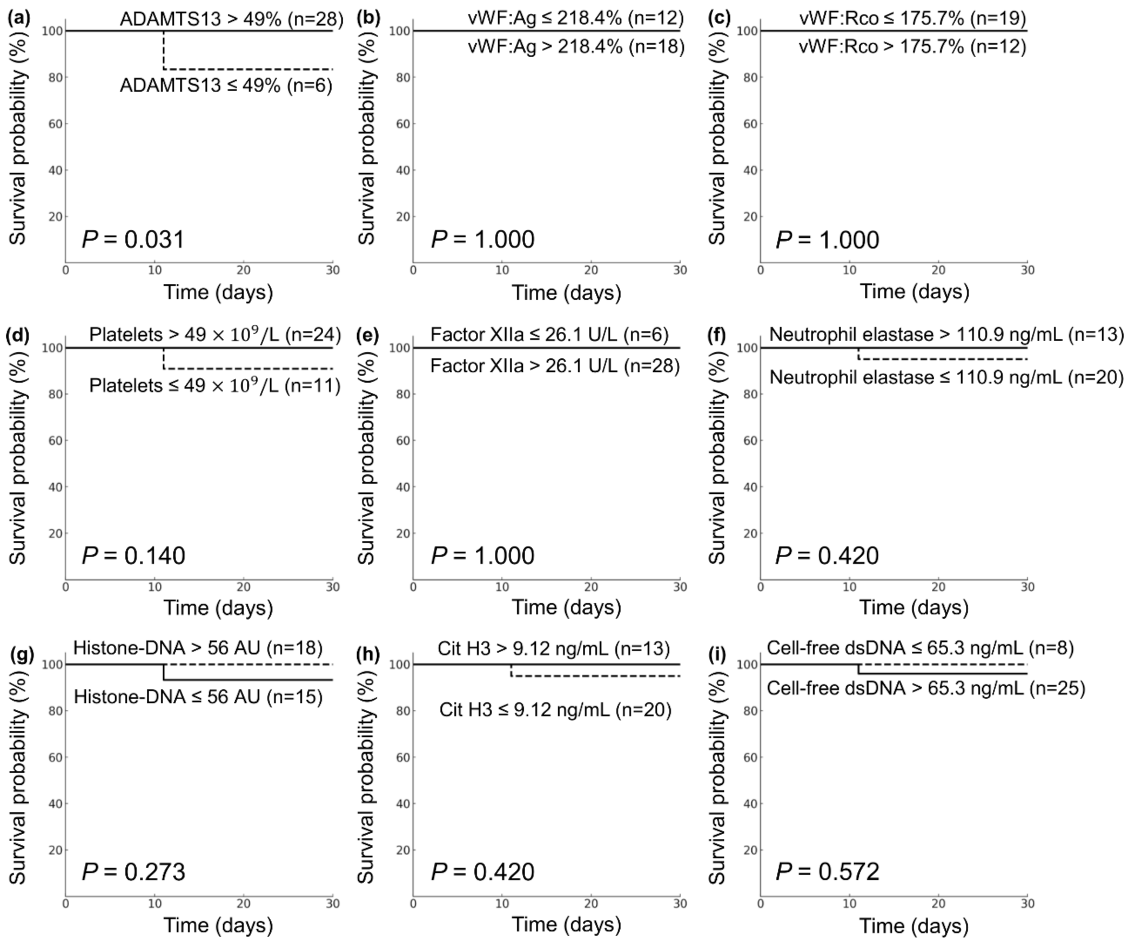

Fig. S3 Kaplan-Meier survival curves in the transplant-related TMA population. Patients were divided into survivors and non-survivors according to the indicated cutoff values of (a) ADAMTS13 activity, (b) vWF:Ag, (c) vWF:Rco, (d) Platelets, (e) Factor XIIa, (f) Neutrophil elastase, (g) Histone-DNA complexes, (h) Cit H3, and (i) Cell-free dsDNA
